# Allele-specific protein binding within the *CD40* locus in human synovial fibroblasts and immune cells

**DOI:** 10.1136/rmdopen-2023-003168

**Published:** 2023-10-25

**Authors:** Larissa Moser, Katerina Laskari, Caroline Ospelt, Miranda Houtman

**Affiliations:** 1 Center of Experimental Rheumatology, Department of Rheumatology, University Hospital Zurich, University of Zurich, Zurich, Switzerland; 2 Division of Rheumatology, Department of Medicine, Karolinska Institutet, Karolinska University Hospital, Stockholm, Sweden

**Keywords:** Polymorphism, Genetic, Arthritis, Rheumatoid, Fibroblasts, B-Lymphocytes, T-Lymphocyte subsets

The region located upstream of *CD40* is one of the rheumatoid arthritis (RA) risk loci. Genetic variants in this risk locus have been found to affect *CD40* mRNA and protein expression.^1 2^ However, most efforts to elucidate how these non-coding variants affect RA susceptibility were performed in monocytes and B cells. Recently, it has been shown that the *CD40* risk locus is also located within open chromatin in synovial fibroblasts (SF), the resident stromal cells of the joints.^3 4^ Although some effects of genetic variants within the *CD40* risk locus have been reported, it remains to be identified whether these variants act similarly in different cell types. Here, we selected putative causal RA variants within the *CD40* risk locus in SF and assessed cell type specificity of these variants.

The *CD40* risk locus contains 11 single nucleotide polymorphisms (SNPs) in linkage disequilibrium (LD; r^2^>0.8) and is located from ~17 Kb of the *CD40* promoter to the first intron of *CD40* (online supplemental file 1A). Based on our previously published data,^4^ the SNPs reside within open chromatin in basal and TNF treated SF. The SNPs rs6074022 (located 6.7 Kb upstream of *CD40*), rs1883832 (located at −1 from the start codon of *CD40*), rs4810485 and rs4239702 (both located within the CD40 transcriptional region) reside within/in the vicinity of regulatory regions (enhancers, promoter) in SF (online supplemental information 1A), highlighting them as putative functional SNPs in SF. Capture HiC analysis suggests that genetic risk variants within the *CD40* locus may influence the regulation of not only *CD40*, but also *NCOA5*, *SLC35C2*, *ELMO2* and/or *ZNF334* (online supplemental file 1B) in SF.

10.1136/rmdopen-2023-003168.supp1 Supplementary data

**Figure 1:**
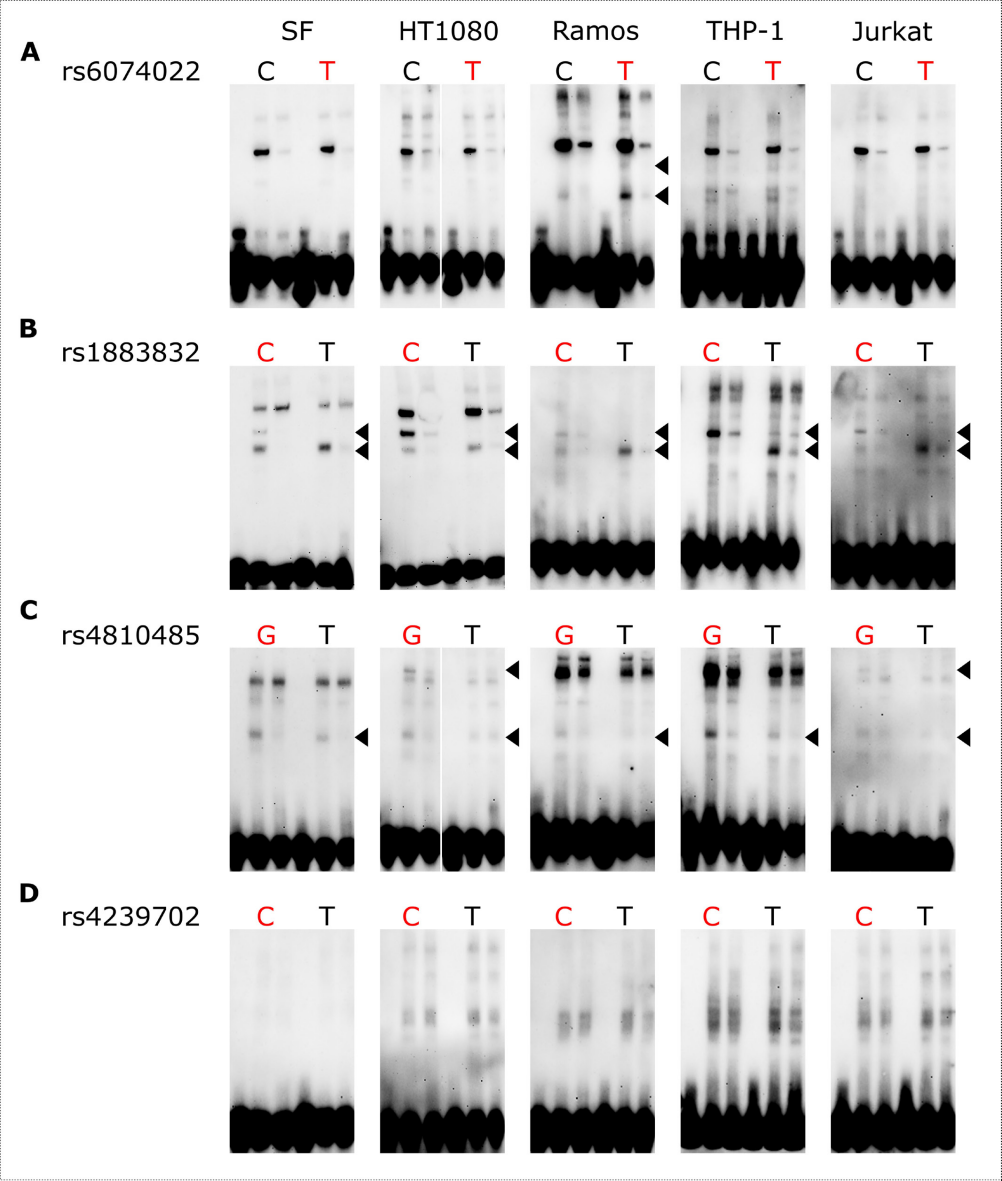
Allele-specific protein binding within the *CD40* risk locus in SF and immune cells. EMSAs were performed with SF, HT1080, Ramos, THP-1 and Jurkat nuclear extracts and biotinylated probes containing rs6074022 (A), rs1883832 (B), rs4810485 (C) and rs4239702 (D). For each allele: lane 1 is probe only, lane 2 is probe with nuclear extract and lane 3 is probe with nuclear extract and excess unlabeled probe. The major/risk allele for each SNP is shown in red. Allele-specific binding is marked with arrowheads. A representative blot of at least three independent experiments is shown (online supplemental file 3). SF, synovial fibroblast.

To identify cell-type specific differences within the *CD40* risk locus, we performed electrophoretic mobility shift assays (EMSAs) with SF, HT1080, Ramos, THP-1 and Jurkat nuclear extracts and biotinylated probes containing the SNPs of interest (online supplemental file 2). We detected allele-specific protein binding for rs6074022 (signal for the rs6074022-T (major/risk) allele) in Ramos cells but not in other cell types, suggesting cell-type specific binding at this motif (figure 1A). Several relevant transcription factors, including interferon regulatory factor (IRF) 1, SP1 and ELF1, were found to bind to the regulatory region at rs6074022 in B cells. In contrast, we observed allele-specific binding for rs1883832 and rs4810485 in all tested cell types. For rs1883832, the C (major/risk) allele showed a specific signal, while the T allele showed a stronger signal than the C allele (figure 1B). Again, IRF1 and SP1 as well as TATA-box-binding protein (TBP) are among the proteins that potentially bind to the regulatory region at rs1883832. The rs4810485-G (major/risk) allele showed a stronger signal compared with the T allele and an additional signal in HT1080 and Jurkat nuclear extracts (figure 1C). The regulatory protein RBPJ, which mediates NOTCH signalling, was identified to control *CD40* expression via rs4810485.^5^ Our data suggest that RBPJ can control the expression of *CD40* via rs4810485 in SF and immune cells. No allele-specific binding for rs4239702 was found in any tested cell type (figure 1D).

10.1136/rmdopen-2023-003168.supp2 Supplementary data

10.1136/rmdopen-2023-003168.supp3 Supplementary data

The sites rs6074022 and rs1883832 were shown to be active under stimulatory conditions (online supplementa file 1B) and are correlated with *CD40* gene expression levels in interferon-γ stimulated cells.^3 6^ This is in line with the potential binding of IRF1 to both sites and points towards a role of interferon-γ in regulating this locus.

Overall, we show that rs6074022 is a putative functional SNP in B cells only and that rs1883832 and rs4810485 have an effect in a broad spectrum of RA-relevant cell types. Our data stress the importance of cell-type-specific effects of genetic variants and provide the basis for future studies to identify the exact mechanisms by which these genetic risk variants in the *CD40* locus are associated with RA.
