# Supplementary material for: Allele-specific protein binding within the CD40 locus in human synovial fibroblasts and immune cells
Source: RMD Open. 2023 Oct 25;9(4):e003168. doi: 10.1136/rmdopen-2023-003168 (PMC10603347; doi:10.1136/rmdopen-2023-003168)
Supplement: Supplementary data [file rmdopen-2023-003168supp001.pdf]

Supplementary Information 1

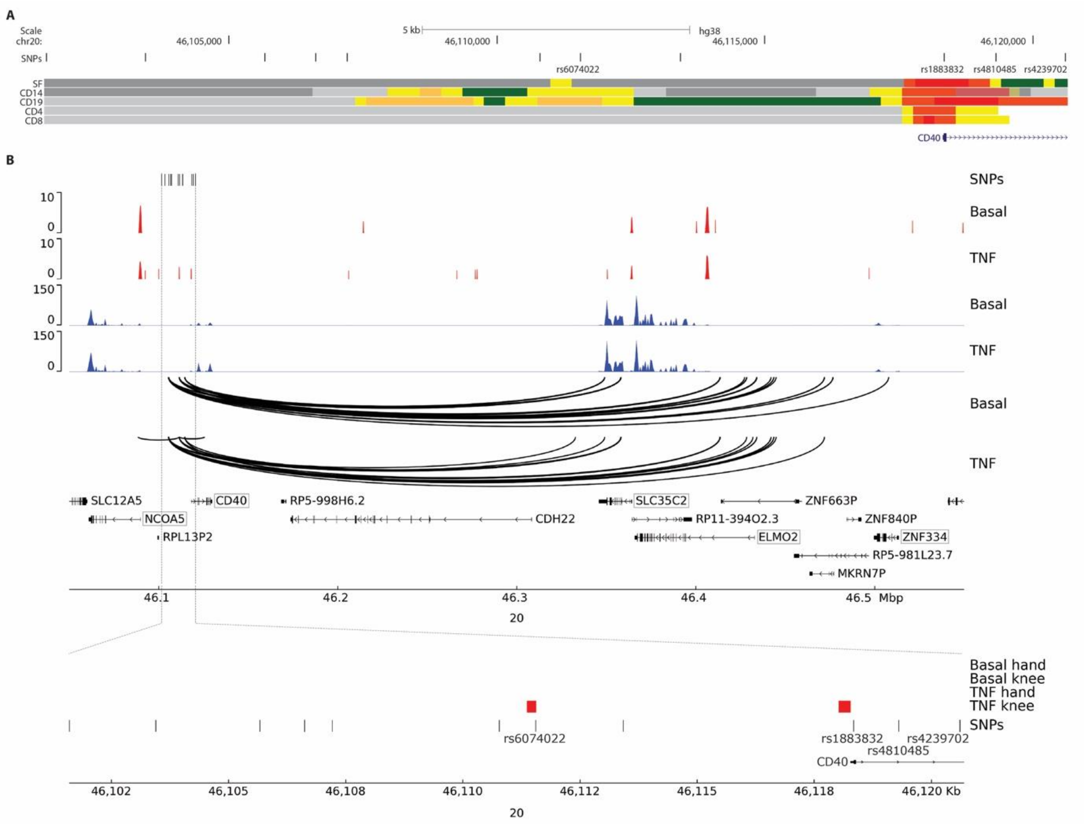

Figure 1. Genome browser view of the *CD40* risk locus in SF. A) ChromHMM annotation in cultured RA SF (1) and immune cells (CD14, CD19, CD4 and CD8 primary cells; Roadmap data). Bright red is active promoter, light red is weak promoter, orange is strong enhancer, yellow is weak/poised enhancer, dark green is transcriptional transition/elongation, light green is weak transcribed, gray is polycomb-repressed, and light gray is repetitive/copy number variation. **B)** Cultured RA SF from hand and knee joints were either treated with TNF (10 ng/ml) for 24h or left untreated (1). The red tracks illustrate ATAC sequencing data, the blue tracks represent RNA sequencing data and the black arcs show capture HiC data. The risk locus with RA risk SNPs (chr20:46,101,606-46,120,612; hg38) is marked with vertical dashed lines. The genes that interact with the risk locus are outlined. The bottom part of the figure shows an enlarged view of the risk locus. Plots were generated with pyGenomeTracks (2). SF: synovial fibroblasts.

References

1. Ge X, Frank-Bertoncelj M, Klein K, McGovern A, Kuret T, Houtman M, et al. Functional genomics atlas of synovial fibroblasts defining rheumatoid arthritis heritability. Genome Biol. 2021;22(1):247.
2. Lopez-Delisle L, Rabbani L, Wolff J, Bhardwaj V, Backofen R, Grüning B, et al. pyGenomeTracks: reproducible plots for multivariate genomic datasets. Bioinformatics. 2021;37(3):422-3.
