# Supplementary material for: Allele-specific protein binding within the CD40 locus in human synovial fibroblasts and immune cells
Source: RMD Open. 2023 Oct 25;9(4):e003168. doi: 10.1136/rmdopen-2023-003168 (PMC10603347; doi:10.1136/rmdopen-2023-003168)
Supplement: Supplementary data [file rmdopen-2023-003168supp002.pdf]

Supplementary Information 2

Methods

Cell culture

Human SF were isolated from synovial tissues obtained from RA patients undergoing joint replacement surgery at the Schulthess Clinic Zurich by mechanical disruption and enzymatic digestion using dispase II (37°C, 1h; 1.5mg/ml). RA patients fulfilled the 2010 ACR/EULAR criteria (1). HT1080 (epithelial), Ramos (B lymphocyte), THP-1 (monocyte) and Jurkat (T lymphoblast) cells were purchased from ATCC. SF and HT1080 cells were cultured in DMEM supplemented with 10% fetal calf serum, 50U ml^{-1} penicillin/streptomycin, 2mM L-glutamine, 10mM HEPES and 0.2% amphotericin B (all from Gibco). Ramos, THP-1 and Jurkat cells were cultured in RPMI-1640 supplemented with 10% fetal calf serum, 50U ml^{-1} penicillin/streptomycin, 2mM L-glutamine, 10mM HEPES and 0.2% amphotericin B (all from Gibco). Cell cultures were maintained at 37°C in a humidified 5% CO_2 incubator. Cultured SF were used between passage 5 to 8.

Nuclear extracts

Nuclear extracts from SF, HT1080, Ramos, THP-1 and Jurkat were prepared using the NE-PER Nuclear and Cytoplasmic Extraction Reagents (Thermo Scientific) according to manufacturer instructions. Halt Protease Inhibitor Cocktail, EDTA-free (Thermo Scientific) was added to Cytoplasmic Extraction Reagent I and Nuclear Extraction Reagent. Protein concentrations were measured using the Pierce BCA Protein Assay Kit (Thermo Scientific).

Biotinylation of oligonucleotides

Single-stranded oligonucleotides of 31bp with the SNP of interest centered in the middle were designed (Table 1), purchased from Microsynth, and biotinylated using the Biotin 3´End DNA Labeling Kit (Thermo Scientific) according to manufacturer instructions. Forward and reverse biotinylated or unlabeled oligonucleotides were mixed in equal molar amounts and incubated for 5 min at 95°C and gradually cooled (-1°C/min) to generate double-stranded probes.

Table 1. Oligonucleotides used for EMSAs.

Oligo ID	Forward (5´-3´)	Reverse (5´-3´)
rs6074022 C	TGCTGAGTGTCCTCACGACATGGCAGACAGC	GCTGTCTGCCATGTCGTGAGGACACTCAGCA
rs6074022 T	TGCTGAGTGTCCTCATGACATGGCAGACAGC	GCTGTCTGCCATGTCATGAGGACACTCAGCA
rs1883832 C	CTGGTCTCACCTCGCCATGGTTCGTCTGCCT	AGGCAGACGAACCATGGCGAGGTGAGACCAG
rs1883832 T	CTGGTCTCACCTCGCTATGGTTCGTCTGCCT	AGGCAGACGAACCATAGCGAGGTGAGACCAG
rs4810485 G	AGGGCTGTAGATTCCGGCCTGAAGCCTGGGC	GCCCAGGCTTCAGGCCGGAATCTACAGCCCT
rs4810485 T	AGGGCTGTAGATTCCTGCCTGAAGCCTGGGC	GCCCAGGCTTCAGGCAGGAATCTACAGCCCT
rs4239702 C	CTTTTAAAACAAAAACCAAGAGCAGGCCTGG	CCAGGCCTGCTCTTGGTTTTTGTTTTAAAAG
rs4239702 T	CTTTTAAAACAAAAATCAAGAGCAGGCCTGG	CCAGGCCTGCTCTTGATTTTTGTTTTAAAAG

Electrophoretic mobility shift assay (EMSA)

EMSAs were performed using the LightShift Chemiluminescent EMSA Kit (Thermo Scientific) according to manufacturer instructions. The binding reactions contained 1x Binding Buffer, 50ng Poly (dI:dC), 2.5% Glycerol, 5mM $MgCl_2$, 0.05% NP-40, 20fmol biotinylated probe, 10µg nuclear extract. For competition experiments, 4pmol of unlabeled probe was added.

Predication of transcription factor binding

Proteins bound in ChIP sequencing experiments from the ENCODE project consortium (2), summarized by HaploReg (3), were used to predict binding of transcription factors to the risk loci.

References

1. Aletaha D, Neogi T, Silman AJ, Funovits J, Felson DT, Bingham CO, et al. 2010 Rheumatoid arthritis classification criteria: an American College of Rheumatology/European League Against Rheumatism collaborative initiative. Arthritis Rheum. 2010;62(9):2569-81.
2. Consortium EP. A user's guide to the encyclopedia of DNA elements (ENCODE). PLoS Biol. 2011;9(4):e1001046.
3. Ward LD, Kellis M. HaploReg: a resource for exploring chromatin states, conservation, and regulatory motif alterations within sets of genetically linked variants. Nucleic Acids Res. 2012;40(Database issue):D930-4.
