# Supplementary material for: Allele-specific protein binding within the CD40 locus in human synovial fibroblasts and immune cells
Source: RMD Open. 2023 Oct 25;9(4):e003168. doi: 10.1136/rmdopen-2023-003168 (PMC10603347; doi:10.1136/rmdopen-2023-003168)
Supplement: Supplementary data [file rmdopen-2023-003168supp003.pdf]

Supplementary Information 3

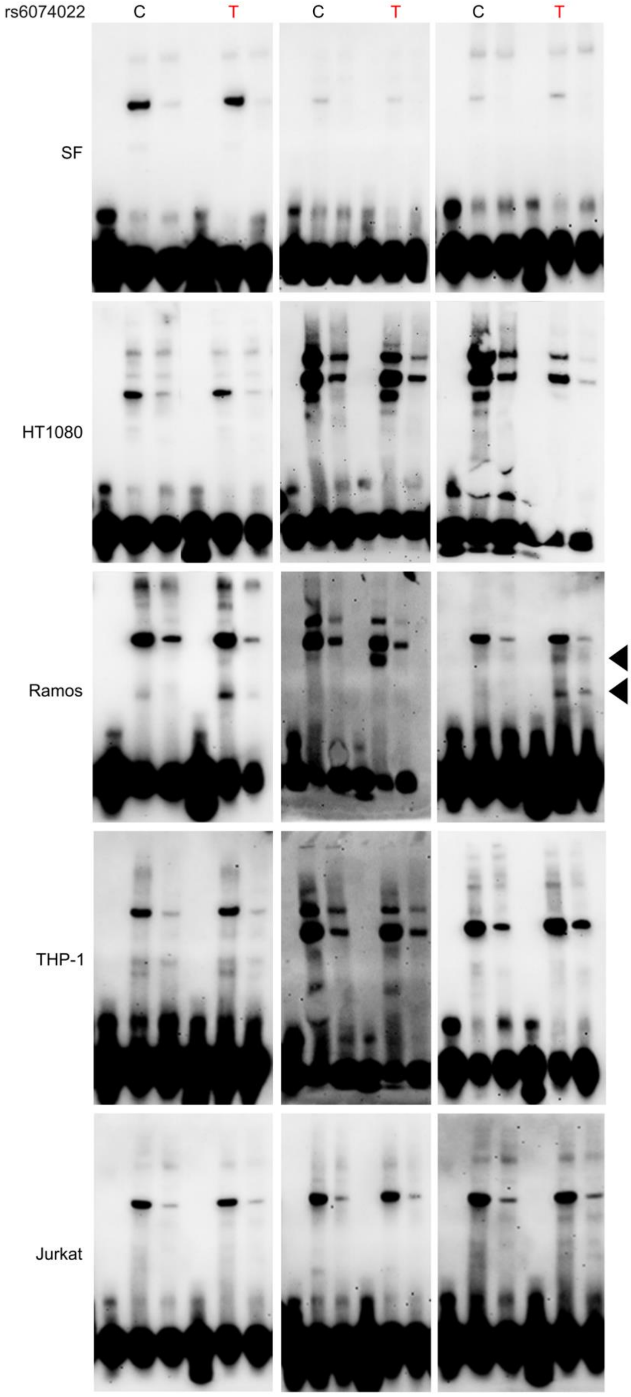

Figure 1. Allele specific protein binding for rs6074022 in SF and immune cells. EMSAs were performed with synovial fibroblast, HT1080, Ramos, THP-1 and Jurkat nuclear extracts and a biotinylated probe containing rs6074022. For each allele: lane 1 is probe only, lane 2 is probe with nuclear extract and lane 3 is probe with nuclear extract and excess unlabeled probe. The major/risk allele for each SNP is shown in red. Allele specific binding is marked with arrowheads.

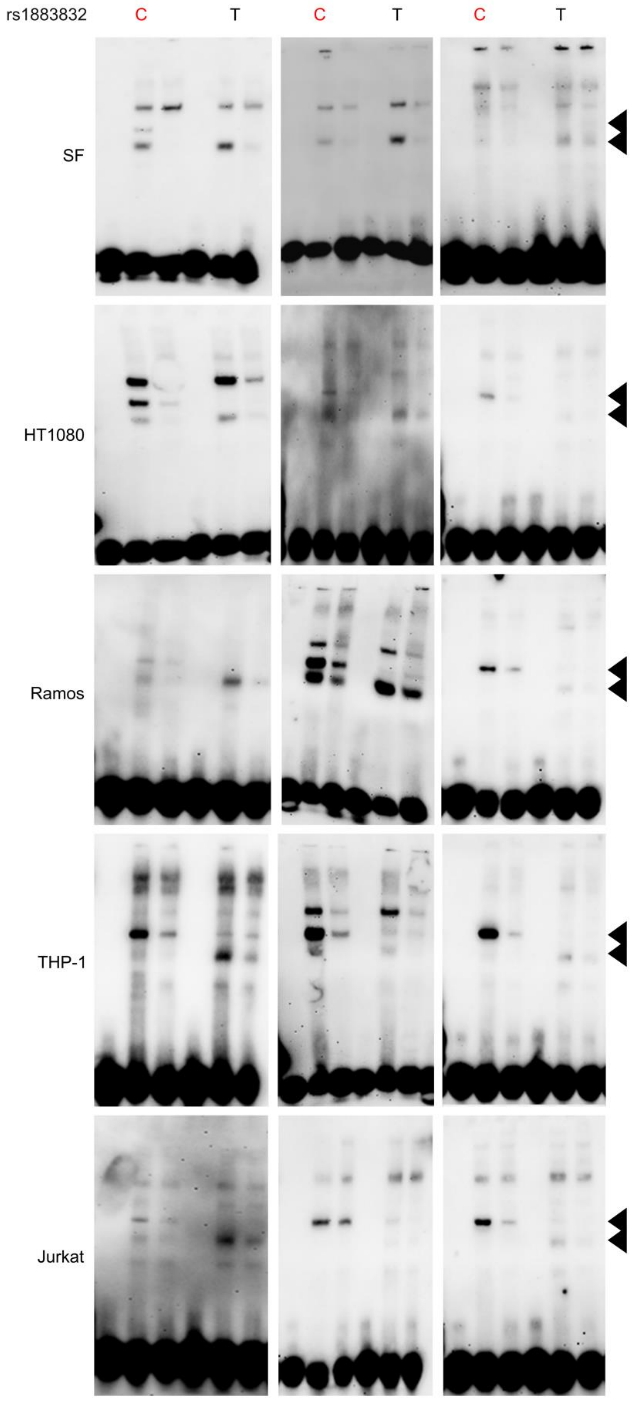

Figure 2. Allele specific protein binding for rs1883832 in SF and immune cells. EMSAs were performed with synovial fibroblast, HT1080, Ramos, THP-1 and Jurkat nuclear extracts and a biotinylated probe containing rs1883832. For each allele: lane 1 is probe only, lane 2 is probe with nuclear extract and lane 3 is probe with nuclear extract and excess unlabeled probe. The major/risk allele for each SNP is shown in red. Allele specific binding is marked with arrowheads.

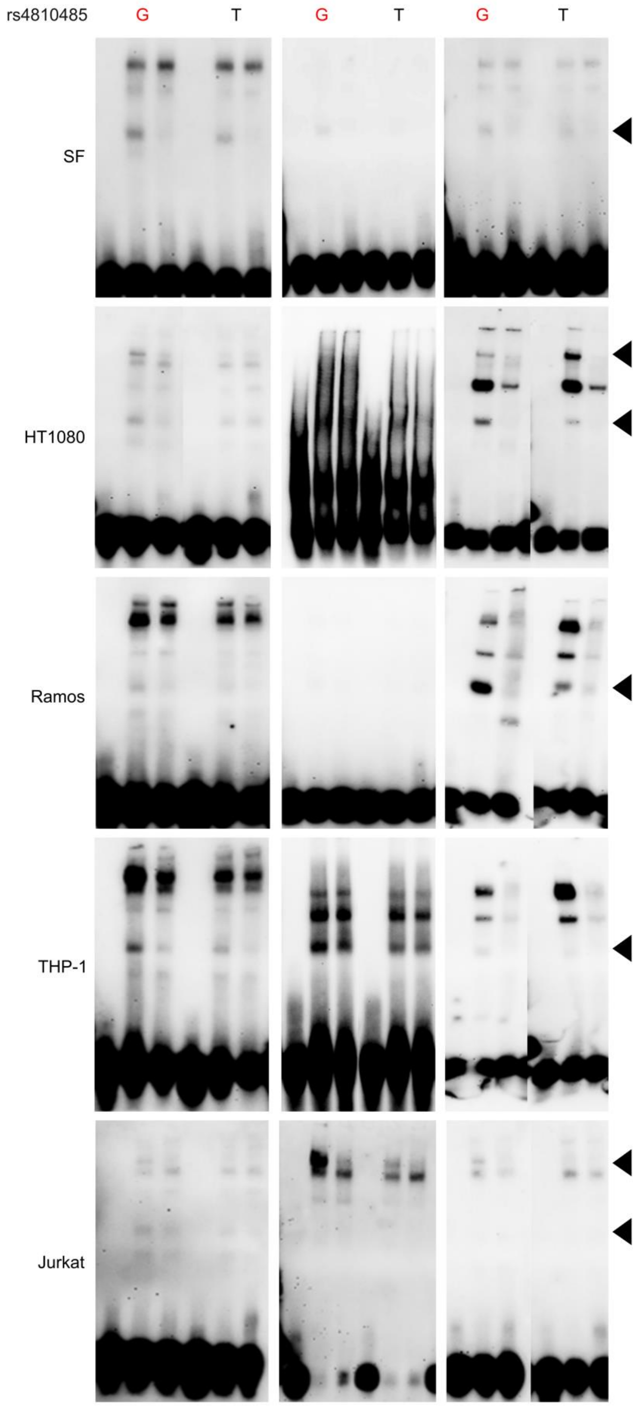

Figure 3. Allele specific protein binding for rs4810485 in SF and immune cells. EMSAs were performed with synovial fibroblast, HT1080, Ramos, THP-1 and Jurkat nuclear extracts and a biotinylated probe containing rs4810485. For each allele: lane 1 is probe only, lane 2 is probe with nuclear extract and lane 3 is probe with nuclear extract and excess unlabeled probe. The major/risk allele for each SNP is shown in red. Allele specific binding is marked with arrowheads.

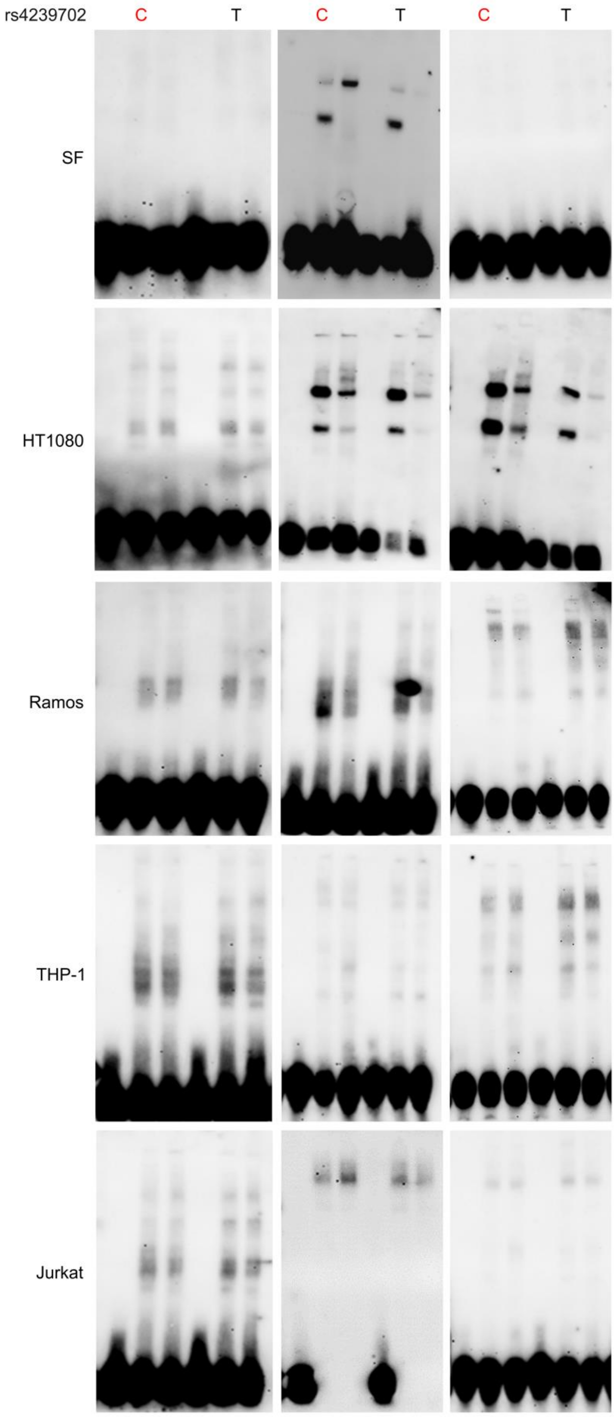

Figure 4. Allele specific protein binding for rs4239702 in SF and immune cells. EMSAs were performed with synovial fibroblast, HT1080, Ramos, THP-1 and Jurkat nuclear extracts and a biotinylated probe containing rs4239702. For each allele: lane 1 is probe only, lane 2 is probe with nuclear extract and lane 3 is probe with nuclear extract and excess unlabeled probe. The major/risk allele for each SNP is shown in red.
